# Genome-wide analysis of transcription factors in prostate cancer

**DOI:** 10.1093/bib/bbaf631.026

**Published:** 2025-12-12

**Authors:** Zimo Zhu, Anne M Gardner, David Wilkes, Alicia Alonso, Cora N Sternberg, Juan Miguel Mosquera, William KM Lai, B Franklin Pugh

**Affiliations:** Department of Molecular Biology and Genetics, Cornell University, Ithaca, NY; Department of Molecular Biology and Genetics, Cornell University, Ithaca, NY; Department of Pathology and Laboratory Medicine, Weill Cornell Medicine, New York, NY; Englander Institute for Precision Medicine, Weill Cornell Medicine, New York, NY; Englander Institute for Precision Medicine, Weill Cornell Medicine, New York, NY; Englander Institute for Precision Medicine, Weill Cornell Medicine, New York, NY; Department of Medicine, Weill Cornell Medicine, New York, NY; Department of Pathology and Laboratory Medicine, Weill Cornell Medicine, New York, NY; Englander Institute for Precision Medicine, Weill Cornell Medicine, New York, NY; Department of Molecular Biology and Genetics, Cornell University, Ithaca, NY; Department of Molecular Biology and Genetics, Cornell University, Ithaca, NY

## Abstract

Prostate cancer (PCa) is one of the most frequently diagnosed cancers in men. PCa growth is dependent on the androgen receptor (AR) which regulates transcription directly as well as through interactions with other transcription factors (TFs). While androgen deprivation therapy is often successful upon progression, a fraction of PCa cases possess constitutively active AR that functions independently of hormonal regulation. Understanding the TF regulatory landscape, especially androgen-dependent versus androgen-independent PCa, is critical to developing better therapeutic strategies. Here we investigated the epigenomic landscape of two prostate cancer cell lines, VCaP (androgen-dependent) and 22Rv1 (androgen-independent), using ATAC-seq to map chromatin accessibility and a high-resolution version of ChIP-seq (ChIP-exo) that characterizes binding of proteins to the genome at base-pair resolution. We identified distinct AR binding between cell lines in response to androgen-treatment, with a subset of AR sites showing strong enhancer enrichment and preferential colocalization with FoxA1 at characteristic spacing, consistent with cooperative AR–FoxA1 regulation. Interestingly, many FoxA1 binding events occurred independently of AR and were enriched in quiescent chromatin, suggesting a context-dependent role for FoxA1. Together, these findings highlight how differential AR–FoxA1 crosstalk and chromatin states contribute to hormone sensitivity versus resistance in PCa.

